# Recurrence of tuberculosis in a low-incidence setting: a retrospective cross-sectional study augmented by whole genome sequencing

**DOI:** 10.1186/s12879-018-3164-z

**Published:** 2018-06-07

**Authors:** Laila Parvaresh, Taryn Crighton, Elena Martinez, Andrea Bustamante, Sharon Chen, Vitali Sintchenko

**Affiliations:** 1 Centre for Infectious Diseases and Microbiology-Public Health, Westmead Hospital, cnr Hawkesbury and Darcy Roads, Westmead, NSW 2145 Australia; 2 NSW Mycobacterium Reference Laboratory, Centre for Infectious Diseases and Microbiology Laboratory Services, NSW Health Pathology, Westmead, NSW 2145 Australia; 3 Centre for Infectious Diseases and Microbiology Laboratory Services, Westmead Hospital, and Sydney Medical School, The University of Sydney, Westmead, NSW 2145 Australia

**Keywords:** Tuberculosis, Recurrent disease, Epidemiology, *Mycobacterium tuberculosis*, Genome sequencing, Genotyping, Disease incidence

## Abstract

**Background:**

The recurrence of tuberculosis (TB) disease in treated patients can serve as a marker of the efficacy of TB control programs. Recurrent disease represents either endogenous reactivation with the same strain of *Mycobacterium tuberculosis* due to non-compliance or inadequate therapy or exogenous reinfection with a new strain. Genotyping or whole genome sequencing (WGS) of *M. tuberculosis* isolates from initial and recurrent cases can differentiate between reinfection and reactivation. This study examined cases of recurrent TB in New South Wales, Australia, using genotyping and WGS.

**Methods:**

Culture-confirmed TB cases diagnosed at least 12 months apart between January 2011 and December 2016 were included. Isolates of *M. tuberculosis* from patients were compared using 24-locus Mycobacterial Interspersed Repetitive Unit Variable Number Tandem Repeat (MIRU-24) typing and WGS.

**Results:**

Eighteen cases of recurrent disease were identified but isolates from only 15 (83%) were available for study. MIRU-24 findings classified 13 (13/15; 87%) as reactivation and two (13%), as reinfection. Sequencing 13 cultivable paired isolates demonstrated 11 reactivations and two reinfections. There was genomic similarity in 10 out of 13 pairs while one case (1/13; 8%) had 12 SNPS differences. Two other cases (2/13;15%) had > 200 SNPs differences and were classified as reinfection. No phenotypic or genomic evidence of drug resistance was observed.

**Conclusion:**

TB control programs can achieve consistently low rates of recurrent disease in low incidence settings. WGS of implicated isolates augments the differentiation between reactivation and reinfection and indicates that the majority of recurrences are due to reactivation rather than reinfection. Predominance of reactivation over reinfection indicates high-quality public health practices and a low risk of local transmission.

**Trial registration:**

This study was approved by the Western Sydney Local Health District (WSLHD) Human Research Ethics Committee (HREC Ref: AU RED LNR/17/WMEAD/190; SSA Ref: LNR SSA/17/WMEAD/191).

## Background

Tuberculosis (TB) remains a major public health threat worldwide with approximately 10.4 million people diagnosed annually [1]. The introduction of Directly Observed Therapy (DOT) has reduced the incidence of this disease and disease recurrence [1–3]. Recurrent disease after a completed treatment is associated with either endogenous reactivation of same strain of *Mycobacterium tuberculosis* or reinfection with a new strain [3, 4]. In low-incidence countries such as Australia, the frequency of recurrent TB is reported as 0.4–6% and has been largely due to endogenous reactivation rather than exogenous reinfection [4–6]. Accurate identification and monitoring of the rate of recurrence is essential as a measure of the success of TB control [7].

Distinguishing between these reactivation or reinfection is typically achieved through molecular genotyping [8]. There are three common conventional methods of molecular genotyping: IS6110-based restriction fragment length polymorphism, spoligotyping or mycobacterial interspersed repetitive units (MIRU) typing [4, 5, 9, 10] More recently, whole genome sequencing (WGS) has been used to interrogate the genome of *M. tuberculosis* for markers of drug resistance [11] and assess the similarity or difference between isolates with the same MIRU profile or epidemiological connections [12].

A study in New South Wales (NSW), the most populous state of Australia, done on culture-confirmed cases diagnosed between 1994 and 2006 and recurrent disease, showed 0.4% rate of recurrent culture-positive disease and attributed 73% of episodes to endogenous reactivation in comparison with a smaller number of exogenous reinfections. This study used spoligotyping and MIRU typing [3]. In this study, we aimed to re-examine rates of reinfection and reactivation of tuberculosis in NSW, in order to update these important key performance indicators for the NSW Tuberculosis Control Program and to examine the added value of WGS in defining reinfection and reactivation of tuberculosis.

## Methods

### Selection of isolates

All *M. tuberculosis* complex isolates recovered from patients in NSW are forwarded to the state Mycobacterium Reference Laboratory (MRL) at the Centre for Infectious Diseases and Microbiology Laboratory Services, Institute of Clinical Pathology and Medical Research, NSW Health Pathology, for confirmatory identification, phenotypic susceptibility testing and genotyping. Resistance testing for first and second line anti-tuberculosis drugs was performed using the Mycobacterial Growth Indicator Tube 960 method (Becton Dickinson, MD, USA). Genotyping has been performed using MIRU since 2007 [13]. All cases of culture-confirmed tuberculosis in NSW between 1st of January 2007 and 31st of December 2016 were de-identified and selected in the analysis. Cases with two or more cultures of *M. tuberculosis* from clinical samples taken at least 12 months apart were selected from laboratory records in MRL. All these cases had apparently completed Directly Observed Therapy Short course (DOTS) [14] for the first episode of their culture positive tuberculosis. The identified isolates were compared by their drug resistance susceptibility profile and MIRU genotype.

### Mycobacterium interspersed repetitive unit (MIRU) typing

The MIRU typing was performed using 24 loci, as described previously [13]. Briefly, DNA extracted from culture isolates was subjected to eight multiplexed PCR’s which include 24 labelled primer sets. Measurement of the resulting PCR products was performed on an ABI 3730 DNA Analyser (Applied Biosystems, Foster City, California) and a 24-digit MIRU profile produced using ABI Peak Scanner Software Version 2.0 (Applied Biosystems, Foster City, California). The isolates were assigned to lineages using the MIRU-VNTR*plus* (http://www.miru-vntrplus.org) online database.

### Whole genome sequencing

Genomic DNA from *M. tuberculosis* isolates was extracted as per standard protocol [11]. Libraries were constructed using the Nextera XT DNA preparation kit (Illumina, San Diego, California) and genome sequencing was performed on NextSeq500 (Illumina) with 2 × 150-bp paired-end chemistry. Sequencing reads were mapped to the reference genome *M. tuberculosis* H37Rv (GenBank NC_000962) and single nucleotide polymorphism (SNP) variants identified using CLC Genomics Workbench version 10.0.1 (Qiagen, Denmark). Reads were processed through the RedDog pipeline (https://github.com/katholt/RedDog) and Snippy v3.1 and screened for mutations associated with drug resistance.

### Differentiation of disease reactivation from reinfection

Cases were judged as endogenous reactivation when *M. tuberculosis* isolates recovered from the first and second episodes of disease had two or less repeat differences in their MIRU profile [12] and less than 10 SNPs difference between their respective genomes [15]. Conversely, greater than 10 SNPs or two or more repeat differences between genomes or MIRU profiles, respectively, was judged as the indication of exogenous reinfection with a different strain. The numbers of cases with documented endogenous reactivation and exogenous reinfection were compared with the total number of culture-confirmed recurrent TB notifications for the period of this study.

The study protocol was approved by the Western Sydney Local Health District (WSLHD) Human Research Ethics Committee (HREC Ref: AU RED LNR/17/WMEAD/190; SSA Ref: LNR SSA/17/WMEAD/191).

## Results

**Fig. 1:**
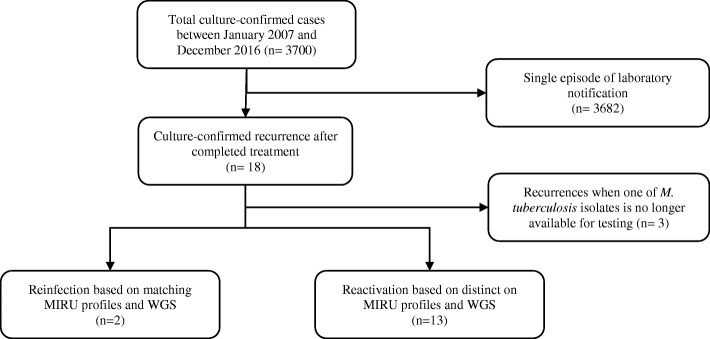
Flow chart showing process of differentiation between reactivation and reinfection

**Fig. 2:**
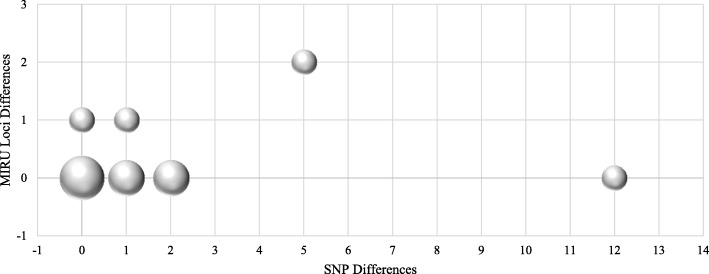
Differences between pairs (*n* = 11 pairs) of *M. tuberculosis* isolates associated with cases of reactivation of tuberculosis based on MIRU (number of MIRU-24 loci) and WGS (number of SNPs). The size of circles is proportional to the number of cases

There were 3700 culture-confirmed cases of TB diagnosed in NSW in 2007–2016. From these cases, 18 cases (18/ 3700; 0.5%) were identified as recurrent disease in patients previously treated for tuberculosis in NSW. MIRU-24 profiles were available for 15 cases (15/18; 83%); *M. tuberculosis* isolates responsible for the first episode of the disease were no longer viable for analysis in three cases (Fig. 1). Based on MIRU-24 analysis and the criteria described above, recurrent tuberculosis was attributed to endogenous reactivation in 13 (13/15; 87%) patients and to exogenous reinfection in 2 (2/15; 13%) patients. Nine patients with reactivation of disease had identical *M. tuberculosis* isolates in their MIRU-24 profiles but four were found to have 1 to 2 loci difference in their MIRU-24 profiles. Isolates associated with exogenous reinfection were different in more than two loci (Fig. 2).

Similarity between bacteria was further examined by whole genome sequencing of 26 *M. tuberculosis* isolates; 11 pairs representing 11 cases of reactivation and two pairs involved in two cases of reinfection as defined by MIRU criteria. Genomic comparison demonstrated less than 5 SNP differences between genomes of isolates of 10 pairs associated with tuberculosis reactivation. Genomes of two *M. tuberculosis* Beijing lineage isolates recovered from one patient with tuberculosis relapse 5 years after the completion of treatment of pulmonary tuberculosis, which were indistinguishable by their MIRU-24 profile, appeared to be different by 12 SNPs. The case was defined as probable reactivation of tuberculosis because longer than average duration of time between episodes of disease may explain the accumulation of SNPs (Fig. 2). The comparison of genomes from isolates from two cases defined by MIRU as exogenous reinfection demonstrated greater than 600 SNP differences between them, confirming the MIRU-24 results. One of the two exogenous re-infected cases was initially infected with *M. tuberculosis* of the European American lineage but had the second episode caused by the Beijing lineage strain. The other case of reinfection had the first episode caused by Beijing lineage strain and subsequent disease by the Delhi/Central Asian (CAS) lineage. The European American lineage was the most common lineage associated with TB reactivation (5/13; 38%), followed by 4 (4/13; 30%) Beijing and 2 (2/13; 15%) Delhi/CAS.

Characteristics of recurrent tuberculosis patients are shown in Table 1. The mean age of patients at the time of first diagnosis was 54 years and 73.3% of patients were male. Most cases were reported affect the lungs (pulmonary TB; 14/15; 93.3%) at initial diagnosis (Table 1). The mean time between the first and second episode of culture-confirmed tuberculosis due to endogenous reactivation was 1 year and 9 months (mean = 1.7 years, median = 1.4) and for two exogenous reinfection cases more than 1 year and 7 months (mean = 1.6 years), respectively. There were neither drug-resistant phenotypes nor genomic markers of drug resistance identified among *M. tuberculosis* isolates in this study. The isolates of *M. tuberculosis* in this study did not show any drug-resistance in phenotype or genotype.

**Table 1:** Culture-confirmed cases of recurrent tuberculosis, 2007–2016

Characteristics	First Episode N (%)	Reactivation N (%)	Reinfection N (%)
Male	11 (73.33)	10 (66.66)	1 (6.67)
Female	4 (26.67)	3 (20.00)	1 (6.67)
Initial smear-positive disease	7 (46.66)	7 (46.66)	0 (0)
Initial smear-negative disease	7 (46.66)	5 (33.33)	2 (13.33)
Initial pulmonary disease	14 (93.33)	12 (80.00)	2 (13.33)
Initial extra-pulmonary disease	1 (6.67)	1 (6.67)	0 (0)
Total genotyped recurrent cases	15 (100.00)	13 (86.67)	2 (13.33)

## Discussion

This study demonstrated a low incidence of recurrent tuberculosis in NSW comparable to rates observed in the previous decade (0.5% in 2007–2016 versus 0.4% in 1996–2006) [3]. Based on both MIRU-24 and WGS analyses, endogenous reactivation was found to be the main source of recurrent disease (87% of cases). Endogenous TB reactivation was common in population aged more than 50 years (mean: 57 years) (*P* < 0.05) and 80% of reactivated tuberculosis cases (*n* = 12; 80%) had pulmonary involvement in their initial presentation (*P* < 0.05) [16].

In contrast to other observations [17, 18], our findings indicated relapses occurring within 2 years after the initial diagnosis and treatment with no temporal difference between reactivation and reinfection. Interestingly, the most common phylogenetic lineage of *M.tuberculosis* associated with recurrent tuberculosis in NSW was European American lineage but not the Beijing or East African Indians strains which have been the most prevalent in Australia in the last decade [12]. Whether this disproportional representation of European American lineage in our dataset reflects more severe and protracted disease [19] warrants further study.

Both MIRU typing and WGS-based genotyping gave concordant results in classifying cases with similar resolution. This can be explained by the predominance of European American lineage in our study set and the relatively low frequency of Beijing lineage isolates (33%) for which MIRU typing could be significantly less discriminatory than WGS [12]. WGS-based differentiation between reactivation and reinfection should be the method of choice for settings with the domination of highly clonal Beijing lineage strains. The absence of resistance defining mutations in genomes of isolates from recurrent disease offered reassurance that unrecognised drug resistance has not played any role [20, 21].

Our findings reconfirmed that reinfection is less likely than reactivation in low-incidence countries for tuberculosis like Australia but can still contribute significantly to the number of cases with recurrent disease. The exogenous reinfection in our study appeared to be responsible for 13% of tuberculosis recurrence, and this rate has been reported to vary from 4 to 33% in various low-incidence countries [4–6, 18, 22, 23].

Several potential limitations of the study are acknowledged. First, our study was limited by a small number of recurrent cases identified in the period of 10 years in one jurisdiction of Australia. Nevertheless, our application of genome sequencing to culture confirmed cases in the low-incidence setting where 72–78% of all tuberculosis diagnoses are confirmed by culture [16] has offered the opportunity to produce high quality data to differentiate reinfection from reactivation. Secondly, there is a possibility of additional cases being missed due to a short follow-up of 2 years. However, our study documented the mean time between the first and second episode of tuberculosis of 15–20 months that should justify the duration of our follow-up. Last, the HIV status of the patients was not assessed despite suggestions that HIV infection can predispose to tuberculosis recurrence following DOTS [24, 25]. The impact of this limitation on our conclusions is likely to be minimal as the frequency of HIV co-infection in patients with newly diagnosed tuberculosis in NSW in 2009–2011 has been reported as 3% [16].

## Conclusion

A jurisdictional tuberculosis control program based on directly observed therapy in a low incidence setting results in persistently low rates of recurrent tuberculosis. High-resolution typing and genome sequencing of *M. tuberculosis* isolates associated with culture-confirmed cases of recurrent tuberculosis enabled the differentiation between reinfection and endogenous reactivation. Predominance of reactivation over reinfection indicates high-quality public health practices and low risk of local transmission. These findings provide important benchmarks for public health policy planning in low incidence countries.

## Abbreviations

CAS: Central Asian
CIDM: Centre of infectious disease and microbiology
DOT: Directly observed treatment
DOTS: Directly Observed Therapy Short Course
EAI: East African Indian
HIV: Human immunodeficiency virus
HREC: Human research ethic committee
MDR-TB: Multi drug resistant tuberculosis
MIRU: Mycobacterial interspersed repetitive unit
MRL: Mycobacterium Reference Laboratory
NSW: New South Wales
RLFP: Restriction fragment length polymorphism
SNP: Single nucleotide polymorphism
TB: Tuberculosis
VNTR: Variable number tandem repeat
WHO: World health organization
